# The Gut–Lung Axis During Ethanol Exposure and a *Pseudomonas aeruginosa* Bacterial Challenge

**DOI:** 10.3390/biomedicines12122757

**Published:** 2024-12-03

**Authors:** Anthony Santilli, Yingchun Han, Hannah Yan, Naseer Sangwan, Gail A. M. Cresci

**Affiliations:** 1 Department of Inflammation and Immunity, Lerner Research Institute, Cleveland Clinic, Cleveland, OH 44195, USA; santila@ccf.org (A.S.);; 2 Microbial Sequencing & Analytics Resource (MSAAR) Facility, Shared Laboratory Resources (SLR), Lerner Research Institute, Cleveland Clinic, Cleveland, OH 44195, USA; 3 Cardiovascular and Metabolic Sciences, Lerner Research Institute, Cleveland Clinic, Cleveland, OH 44195, USA; 4 Cleveland Clinic Lerner College of Medicine of Case Western Reserve University, Cleveland, OH 44195, USA; 5 Department of Gastroenterology, Hepatology and Nutrition, Digestive Disease Institute, Cleveland Clinic, Cleveland, OH 44195, USA

**Keywords:** gut microbiome, gut–lung axis, ethanol exposure, pulmonary infection

## Abstract

**Background:** Susceptibility to and severity of pulmonary infections increase with ethanol consumption. We have previously shown that ethanol-induced changes in the gut microbiome disrupt gut homeostasis, allowing for the translocation of proinflammatory mediators into the circulation and eliciting an immune response in the lung. Additionally, targeting the gut with butyrate supplementation not only rescues ethanol-induced disruptions to gut health but also reverses aspects of immune dysregulation in the lungs. Here, we assessed the impact of this connection on a subsequent infectious challenge. **Methods**: To assess if ethanol-induced alterations to the gut microbiome could also impact the host response to a pulmonary infectious challenge, we employed a chronic-binge ethanol-feeding mouse model followed by a nasal instillation of *Pseudomonas aeruginosa*. **Results**: In addition to altering gut microbiome composition and metabolism, ethanol consumption also disrupted the local immune response as demonstrated by suppressed cecal SIgA levels, a decreased presence of CD3^+^CD8a^+^ cytotoxic T cells in the proximal colon mucosa, and depleted CD3^+^CD8a^+^ T cells and CD11c^+^CD8a^+^ dendritic cells in the mesenteric lymph nodes. Circulatory Ly6G^+^CD11b^+^ neutrophils increased, indicating a systemic change in immune-cell presence with ethanol exposure. Ethanol exposure increased CD11c^+^CD64^+^ macrophages and Ly6G^+^CD11b^+^ neutrophils in the lungs, with neutrophil populations being further exacerbated during a bacterial challenge with *Pseudomonas aeruginosa*. Lipocalin 2, a marker of oxidative stress, was also elevated with ethanol consumption, though not with infection. **Conclusions**: These data suggest that ethanol-induced changes in the gut microbiome and immune environment are linked to dysfunctional immune responses in the intestine, blood, and the lungs, compromising the pulmonary immune response during an infectious challenge in mice.

## 1. Introduction

The gut microbiome is important for maintaining health. The gut microbiota rely upon the environment and energy resources available within the gut lumen to fuel their metabolic processes. Microbial metabolites such as short-chain fatty acids (SCFAs) can be utilized by the host to support gut health, barrier functions, and immune responses. A “healthy” gut microbiome is characterized by the domination of two bacterial phyla, Firmicutes and Bacteroidetes, and reduced abundances of taxa within these phyla and overabundances of potentially pathogenic taxa have been linked to various illnesses [1]. Manipulations of the gut environment, either through alterations to microbial populations with dietary prebiotic- or probiotic-containing supplements, fecal microbiota transplants, or supplementation with bacterially derived metabolites, have also been shown to mitigate factors related to chronic inflammatory intestinal conditions and have, therefore, been suggested as potential therapeutics [2,3,4,5]. Factors that alter the composition or function of the microbiome, such as diet, antibiotic usage, and age, have been shown to influence local gut immune factors as well as the immune state of discrete organ systems [1,6,7]. Such associations have given rise to investigations into various gut axes (gut–liver, gut–lung, etc.) to describe the mechanisms by which facets of gut health can communicate with or influence outside organ systems and vice-versa.

Chronic alcohol consumption is a factor that disrupts gut microbiome homeostasis as well as immune responses in several organs, such as the gastrointestinal tract [8,9], liver [9,10,11], lungs [1,12,13,14], and central nervous system [6,15,16]. Chronic alcohol use has been shown to alter the abundance of several gut commensal bacteria and their metabolic byproducts both in human studies [17,18] and in pre-clinical animal models [19,20]. Short-chain fatty acids (acetate, propionate, and butyrate) are beneficial gut microbial metabolites. Butyrate has many beneficial biological roles in the gut, where it supports the gut barrier integrity by maintaining intestinal tight junctional protein complexes and modulates immune and inflammatory responses [7]. The loss of these defenses allows antigens such as bacteria and endotoxins, normally contained within the gut lumen, to escape into systemic circulation through the vascular and/or lymphatic systems and initiate immune responses and tissue damage throughout the body.

Our group has previously shown that proinflammatory mediators escaping the gut during ethanol consumption can influence innate immune responses within the lung [14], characterized by elevated neutrophils and oxidative stress. Additionally, oral supplementation with butyrate restored ethanol-induced changes in gut immune responses and lessened lung immune responses, suggesting a causative link between the gut–lung axis during ethanol exposure [14].

Clinically, alcohol-use disorder is linked to an increased susceptibility to and severity of pulmonary bacterial infections [21,22,23,24], and is casually linked as a risk factor for SARS-CoV-2 infection [12,21]. Additionally, mouse models of ethanol exposure have described alterations to pulmonary T cell and neutrophil abundances and their activation during subsequent fungal [13] or bacterial challenges [1]. As alcohol alters the gut microbiome, leading to local immune disruption, and because such changes are associated with immune-cell infiltration and activation in the lungs, we hypothesized that ethanol’s impact on the gut–lung axis might hinder the ability of the pulmonary immune system to prevent or respond to an acute infectious challenge. We utilized the Lieber DeCarli chronic-binge ethanol-feeding protocol, which mimics acute-on-chronic alcoholic liver injury in patients by inducing liver injury, inflammation, and steatosis [25]. Additionally, this model has previously been shown to impact inflammatory markers in the lungs [26]. *P. aeruginosa* is a common cause of hospital- and intensive care unit (ICU)-acquired pneumonia [27], and AUD is a major risk factor for community-acquired pneumonia with *P. aeruginosa* and other Gram-negative organisms. Therefore, to model *P. aeruginosa*’s ability to influence acute pulmonary immune responses with a background of chronic-binge ethanol exposure, *P. aeruginosa* was introduced via nasal inoculation 24 h after the ethanol binge.

## 2. Materials and Methods

### 2.1. Chronic-Binge Ethanol-Feeding Model with and Without P. aeruginosa Nasal Inoculation

Female C57BL/6 mice were obtained from Jackson Labs (Bar Harbor, ME, USA) at 10–11 weeks of age. Mice were randomized to receive the chronic-binge ethanol Lieber DeCarli feeding model [25], consisting of 10 days chronic 5% ethanol incorporated into a liquid diet followed by a 5 g/kg “binge” oral gavage on the 11th day. Control mice were pair-fed an isocaloric diet where maltose was substituted for ethanol. To investigate acute immune responses in the gut–lung axis to a bacterial challenge from the background of chronic-binge ethanol exposure, mice intranasally received either 10^6^ colony forming units (CFUs) of *Pseudomonas aeruginosa* (*Pseudomonas aeruginosa* FP; ATCC 10145GFP) or saline 24 h after the ethanol binge. This produced the following four separate treatment groups: pair-fed saline (PF-S), ethanol-fed saline (EF-S), pair-fed *P. aeruginosa* (PF-Pseudo), and ethanol-fed *P. aeruginosa* (EF-Pseudo). Mice were euthanized 24 h after the nasal inoculation (48 h post ethanol binge), blood was collected from the inferior vena cava, and tissue was dissected and stored for later analyses.

A separate group of mice received the same ethanol-feeding model, but were euthanized 6 h after the ethanol binge and did not receive an intranasal administration of *P. aeruginosa* or saline. This allowed for the following two additional groups: pair-fed (PF) and ethanol-fed (EF).

### 2.2. Shotgun Metagenomics Sequencing, Bioinformatics, and Statistical Analyses of Bacterial gDNA from Cecal Contents

Bacterial gDNA extraction, sample preparation, and sequencing were performed as previously described [14]. Briefly, a Zymo Research Quick DNA Fecal/Soil Microbiome MiniPrep Kit was used to extract gDNA from cecal contents following the manufacturer’s instructions.

Reads were assessed through low quality filtering and host-derived reads were excluded using the reference human genome (version GRCh38p.14) via BBMap software (https://sourceforge.net/projects/bbmap/, accessed on 15 October 2024; version 34.62). Trimmed reads were further processed using Metaphlan (Ver. 1) and Humann (ver. 2).

The DAtest package (https://github.com/Russel88/DAtest/wiki/usage#typical-workflow, accessed on 15 October 2024; version 2.8.0) was utilized for differential abundance benchmarking. LefSeq and metagenomeSeq were selected based on this analysis. Statistical significance was assessed throughout, and the Benjamini and Hochberg method was used to control the false discovery rate. In R (version 4.1.2), genera and species abundance were assessed via linear regression and Wilcoxon tests against metadata variables [14]. Individual features (pathways) were assessed using Tukey’s honest significance (HSD) test.

### 2.3. Isolation of Immune Cells from Mesenteric Lymph Nodes (MLNs)

Immune cells from the MLN were isolated as described by Qiu et al. [28]. Briefly, the MLN chain was identified and located with the last node near the cecum, and the MLN chain along the colon was dissected. MLNs were isolated from the mesenteric fat and disassociated using a 70 µM strainer and 3 mL syringe plunger. Cells were then pelleted and resuspended in PBS and used for the flow cytometry analysis (see below).

### 2.4. Isolation of Immune Cells from Whole Blood

Blood was collected from the inferior vena cava with anticoagulation tubes following anesthesia. Red blood cells were lysed twice using an ACK Lysing Buffer (GIBCO, Waltham, MA, USA). Immune cells were harvested by centrifugation, pelleted, and resuspended in PBS. Immune cells were used for the flow cytometry analysis (see below).

### 2.5. Isolation of Lung Immune Cells

Immune cells were isolated from lung tissue as previously described [14]. Briefly, after euthanasia, lungs were perfused with cold PBS, then dissected. Tissue was then digested with collagenase, followed by incubation at 37 °C in an orbital shaker, and finally disassociated using a 70 µM strainer.

### 2.6. Flow Cytometry Analysis of MLN, Lung, and Whole-Blood Immune Cells

Isolated immune cells were washed, stained, and blocked as described previously [14]. MLN and whole-blood immune cells were stained using the following fluorescence-conjugated antibodies: anti-CD45-APCFire 810, anti-CD11c-BV650, anti-CD3-FITC, anti-CD4-BUV805, anti-CD8a-BUV615, anti-FoxP3-PE, anti-CD103-BV785, anti-Ly6G-PE-Cy7, and anti-CD11b-eFlour450. Lung immune cells were stained using the following fluorescence-conjugated antibodies: anti-CD45-AF700, anti-SiglecF-BV421, anti-CD11b-BV605, anti-CD11c-AF488, anti-CD64-AF350, and anti-Ly6G-PeCy7. Following staining, samples were washed 3 times and cells were acquired using a Sony ID 7000 Spectral Cell Analyzer (San Jose, CA, USA). The analysis of flow cytometry results was performed using FloJo ver. 10.8 (BD Life Sciences, Sparks, MD, USA).

### 2.7. RT-qPCR

Tissue cDNA from mRNA and bacterial gDNA were prepared as previously described [14] and used for real-time PCR amplification using PowerUp SYBR green master mix (Applied Biosciences, Waltham, MA, USA) and 1 µM primers (Table 1 and Table 2). The relative expression was assessed using the comparative threshold (CT) method using internal controls with either glyceraldehyde 3-phosphate dehydrogenase (GAPDH) for tissue cDNA or universal bacterial 16s for bacterial gDNA. Data were shown as the mean ± standard deviation.

### 2.8. Cecal Secretory IgA (SIgA) Enzyme-Linked Immunoassay (ELISA)

Cecal contents were extracted, and supernatants were prepared and diluted as described previously [14]. SIgA levels were quantified using a Mouse IgA uncoated ELISA Kit (Invitrogen, Waltham, MA, USA) according to the manufacturer’s instructions. A BioTek Synergy H1 Microplate Reader and BioTek software ver. 3.12 (Agilent Technologies, Santa Clara, CA, USA) were utilized for the ELISA plate analysis.

### 2.9. Immunohistochemical Analysis of Proximal Colon Tissue

Dissected proximal colon tissue preserved in optimal cryogenic temperature (OCT) media was sectioned and used for the immunohistochemical staining of CD3^+^, CD8a^+^, and CD3^+^CD8a^+^ co-positive immune cells. Three images were taken per tissue section and semi-quantified using Image-Pro Plus 7 (Media Cybernetics, Silver Springs, MD, USA) to measure the total area of the tissue and positive staining.

### 2.10. Statistical Analysis

Statistical analyses were performed using GraphPad Prism^®^ 10 ver. 10.1.2 (San Diego, CA, USA) and data were shown as the mean ± standard error of the mean. The statistical significance between treatment groups was identified using two-tailed *t*-tests with a threshold for significance of *p* ≤ 0.05.

## 3. Results

### 3.1. Ethanol Exposure Alters Gut Microbiota, Suppressing Butyrate-Producing Members and Altering Bacteria Metabolic Pathways

**Figure 1:**
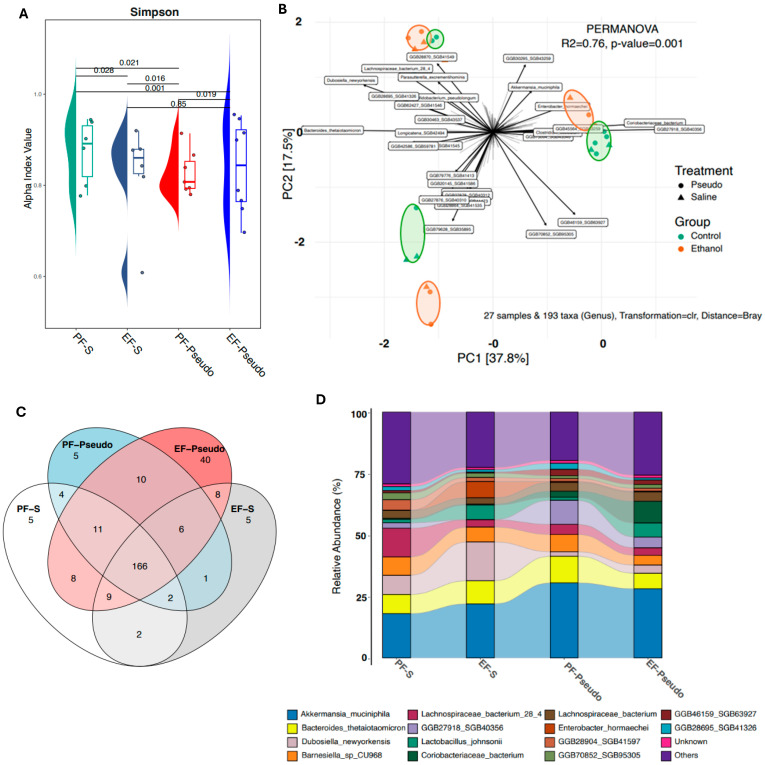
Alterations to cecal microbiome induced by ethanol consumption. Cecal gDNA contents underwent shotgun metagenomic sequencing analysis. (**A**) Simpson index alpha diversity between treatment groups. (**B**) PCoA graph depicting Bray–Curtis dissimilarity. Ellipses were used to visually highlight differences between ethanol-fed and pair-fed groups and do not represent any statistical analysis. (**C**) Venn diagram displaying taxa overlap between treatment groups. (**D**) Stacked graph depicting relative abundance of top species identified in cecal contents. PF-S: control diet + intranasal saline; EF-S: ethanol diet + intranasal saline; PF-Pseudo: control diet + intranasal *P. aeruginosa*; EF-Pseudo: ethanol diet + intranasal *P. aeruginosa*.

Ethanol consumption disrupts the composition and metabolic processes of the gut microbiome and can impair host immune functions within the intestine. It is thought that intestinal immune disruptions are not localized to the intestine and can affect other organ systems such as the hepatic, pulmonary, and nervous systems. To identify ethanol-associated shifts in gut microbial populations and metabolism, bacterial gDNA from mouse cecal contents were assessed via shotgun metagenomic sequencing. The Simpson index showed a significant decrease in alpha diversity in all cohorts compared with the PF-S cohort (Figure 1A). A principal coordinate analysis (PCoA) graph generated using a Bray–Curtis analysis showed three distinct clusters (Figure 1B). Within each cluster, pair-fed (PF; orange) and ethanol-fed (EF; green) mice displayed separated groupings, indicating divergent microbial compositions based on diet. A Venn diagram produced using the Bray–Curtis analysis showed the number of unique and shared taxa among treatment groups (Figure 1C). A total of 166 species were shared among all four treatments, with the EF-Pseudo group displaying the largest number of unique species compared with the other treatment groups. To assess changes in the presence of specific species, relative abundance graphs were generated, which indicated that each treatment group housed a unique cecal microbe composition (Figure 1D). Although these findings highlighted the effect of ethanol on the microbiome, interestingly, *P. aeruginosa* inoculation alone altered the microbial presence in the gut, as highlighted in Figure 1B,D.

**Figure 2:**
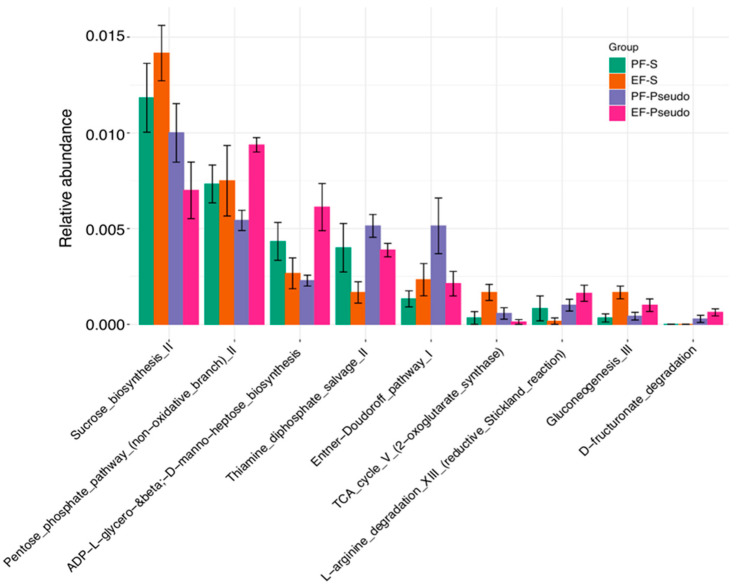
Alterations to microbial metabolic pathways in murine cecal contents identified using shotgun metagenomic sequencing. PF-S: control diet + intranasal saline; EF-S: ethanol diet + intranasal saline; PF-Pseudo: control diet + intranasal *P. aeruginosa*; EF-Pseudo: ethanol diet + intranasal *P. aeruginosa*. Differential abundance analysis among all groups was performed using metagenomeSeq followed by Tukey’s honest significant difference (HSD) test to assess if each of the features (pathway) that were predicted in the differential abundance analysis could individually differentiate the pathways.

These changes in microbial composition coincided with alterations to several metabolic processes (Figure 2). Along with an elevation in microbial gene expression for the citric acid cycle (TCA V pathway), several pathways involved in the biosynthesis of mono- and disaccharides (e.g., sucrose and glucose) were elevated in the ethanol-fed mice compared with the pair-fed mice. Additionally, the thiamine diphosphate salvage system microbial gene expression was decreased in the ethanol-fed mice compared with the pair-fed mice. This upregulation in sugar biosynthesis suggests that the bacteria may have been deprived of the complex carbohydrates they prefer for their metabolism. Interestingly, thiamine plays a key role in many metabolic processes employed by bacteria, including those involved in glycolysis and the TCA V pathway.

**Figure 3:**
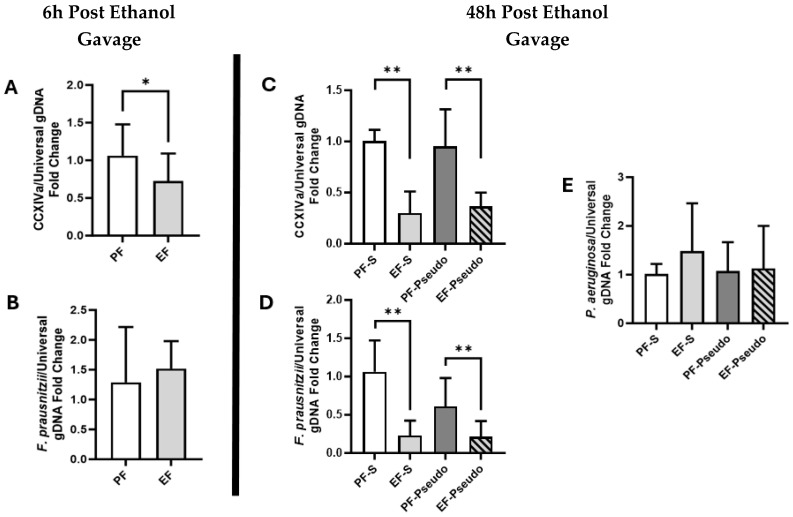
Loss of butyrate-producing taxa during ethanol exposure. (**A**) CCXIVa gDNA expression in cecal contents at 6 h post ethanol binge via qRT-PCR. (**B**) *F. prausnitzii* gDNA expression in cecal contents at 6 h post ethanol binge via qRT-PCR. (**C**) CCXIVa gDNA expression in cecal contents via qRT-PCR at 48 h post ethanol binge and ±*P. aeruginosa* nasal instillation. (**D**) *F. prausnitzii* gDNA expression via qRT-PCR in cecal contents at 48 h post ethanol binge and ±*P. aeruginosa* nasal instillation. (**E**) *P. aeruginosa* gDNA expression via qRT-PCR in cecal contents at 48 h post ethanol binge and ±*P. aeruginosa* nasal instillation. There were 6–12 mice per treatment group. PF: control diet; EF: ethanol diet; PF-S: control diet + intranasal saline; EF-S: ethanol diet + intranasal saline; PF-Pseudo: control diet + intranasal *P. aeruginosa*; EF-Pseudo: ethanol diet + intranasal *P. aeruginosa*. * *p* < 0.05; ** *p* < 0.01.

Butyrate, a fermentation byproduct of the gut microbiome, is important for maintaining gut immune and intestinal barrier functions. Due to butyrate’s important biological roles, we assessed selected butyrate-producing bacteria in cecal contents using RT-qPCR, including Clostridium Cluster XIVa [29] and *F. prausnitzii* [30]. The Clostridium Cluster XIVa (CCXIVa) gDNA expression was reduced in the ethanol-fed mice 6 h post ethanol binge compared with the pair-fed mice (Figure 3A; *p* = 0.044). This finding persisted 48 h after the ethanol binge in mice with and without the administration of *P. aeruginosa* compared with their respective pair-fed controls (Figure 3C; *p* = 0.009 and 0.04, respectively). *F. prausnitzii*, an important anti-inflammatory and butyrate-producing commensal bacteria, was reduced in a similar manner 48 h post ethanol exposure (Figure 3D; *p* = 0.001 and 0.008, respectively), though not at 6 h post-binge (Figure 3B).

We also assessed for *P. aeruginosa* in cecal contents to ensure that the intranasal inoculation of *P. aeruginosa* was not inadvertently entering the intestine and acting as a confounding factor for any gut immune changes. No elevation of *P. aeruginosa* was identified via RT-qPCR in the cecal contents (Figure 3E).

### 3.2. Ethanol-Induced Disruptions to the Gut Immune Environment Persist at 48 h Post Exposure

**Figure 4:**
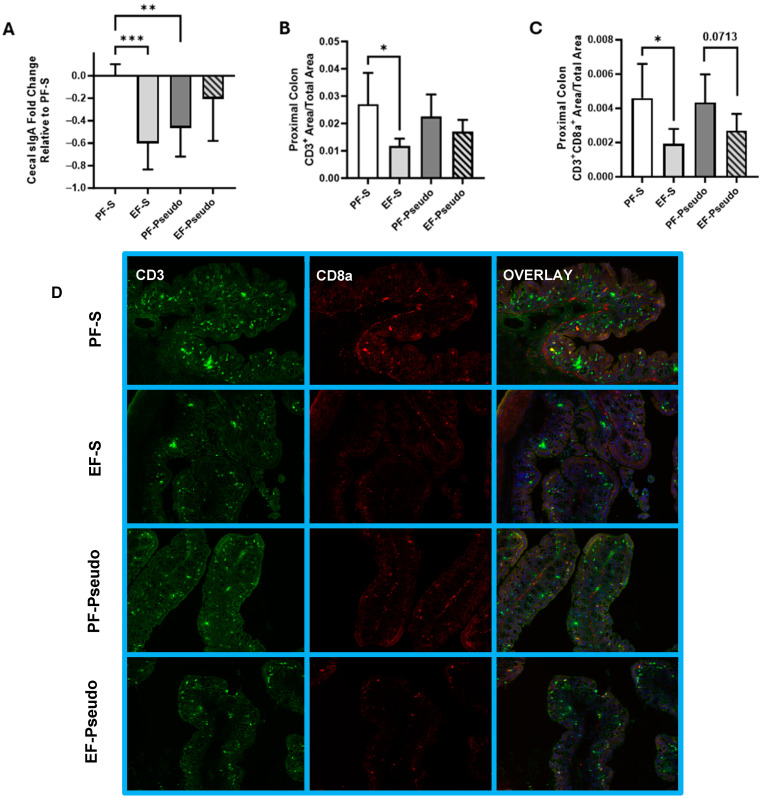
Ethanol feeding reduces cecal SIgA levels and T cells in the proximal colon. (**A**) SIgA concentrations in mouse cecal contents as measured by ELISA. Data are presented as a fold change relative to PF-S mice. (**B**) The abundance of positive staining (area) for CD3^+^ cells in the proximal colon was assessed using immunohistochemical analysis. (**C**) The abundance of positive staining (area) of CD3^+^CD8a^+^ co-positive cells in the proximal colon was assessed using immunohistochemical analysis. (**D**) Representative images using a 20× field for proximal colon tissue stained for CD3 (green), CD8a (red), and DAPI (blue), with 4–6 mice per treatment group. PF-S: control diet + intranasal saline; EF-S: ethanol diet + intranasal saline; PF-Pseudo: control diet + intranasal *P. aeruginosa*; EF-Pseudo: ethanol diet + intranasal *P. aeruginosa*. * *p* < 0.05; ** *p* < 0.01; *** *p* < 0.001.

Alterations to the gut microbiome composition and function can have an outsized influence on the host, both locally and systemically. As butyrate is a key mediator in the gut immune function, we sought to identify if the loss of butyrate-producing commensals during ethanol consumption compromised important aspects of the gut immune system. Secretory IgA (SigA) is secreted by B Cells into the intestinal lumen or is bound to the mucosa, forming a defense barrier that prevents microorganisms and pathogenic microbial factors from interfacing with and potentially bypassing the intestinal epithelium. In testing for SigA in cecal contents, a reduction in SigA was identified in the EF-S cohort compared with the PF-S mice (Figure 4A; *p* = 0.0002). Additionally, the PF-Pseudo group also had depleted cecal SigA levels compared with the PF-S group (Figure 4A; *p* = 0.0015), suggesting that a nasal inoculation of bacteria alone could disrupt intestinal immune homeostasis.

Intestinal immune disruption was also noted within the lamina propria of the proximal colon. Immunohistochemical staining revealed an altered presence of T cells. Both CD3^+^-positive T cells (Figure 4B,D; *p* = 0.021) and CD3^+^CD8a^+^ co-positive cytotoxic T cells (Figure 4C,D; *p* = 0.029) were depleted in ethanol-fed mice that received intranasal saline compared with pair-fed controls. Ethanol-fed mice administered *P. aeruginosa* displayed a similar trend, though it was not statistically significant (Figure 4B,C). Taken together with the loss of SigA, these data suggest an enhanced opportunity for bacteria to reach the intestinal epithelium during ethanol consumption.

**Figure 5:**
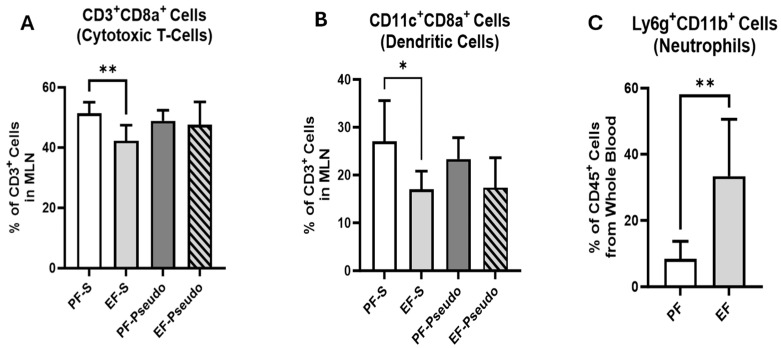
Ethanol treatment suppresses immune-cell presence in MLN but elevates neutrophils in plasma. (**A**) The percentage of CD3^+^CD8a^+^ cells present in the MLN was assessed using flow cytometry. (**B**) The percentage of CD11c^+^CD8a^+^ cells present in the MLN was assessed using flow cytometry. (**C**) The percentage of Ly6G^+^CD11b^+^ cells present in whole blood was assessed using flow cytometry. There were 4–6 mice per treatment group. PF: control diet; EF: ethanol diet; PF-S: control diet + intranasal saline; EF-S: ethanol diet + intranasal saline; PF-Pseudo: control diet + intranasal *P.* *aeruginosa*; EF-Pseudo: ethanol diet + intranasal *P. aeruginosa*. * *p* < 0.05; ** *p* < 0.01.

The intestinal lymph is a potential route for bacterial substances to circumvent gut defenses to reach systemic circulation via the drainage of the thoracic lymph duct into the subclavian vein [31,32,33]. We previously showed an elevated presence of Enterococci in gut-associated lymphatic tissue (GALT) and elevated endotoxin in the plasma of ethanol-fed mice [14]. MLNs collect bacteria and antigens, including endotoxins, from the intestine and GALT before they reach the thoracic duct. Thus, a reduced abundance of immune cells patrolling this pathway could allow increased proinflammatory mediators to enter the circulation. Using flow cytometry, we identified a reduced percentage of both CD3^+^CD8a^+^ cytotoxic T cells and CD11c^+^CD8a^+^ dendritic cells in the MLNs of the EF-S mice compared with the PF-S control mice (Figure 5A,B; *p* = 0.008 and 0.045, respectively); however, this association was not observed in *P. aeruginosa*-infected mice. Both decreased immune-cell abundance in MLNs and increased plasma endotoxin levels, as previously identified [14], could elicit a systemic immune response capable of affecting other organ systems such as the lungs. To identify the potential for a systemic response, we assessed Ly6G^+^CD11b^+^ neutrophils in circulating whole blood 6 h following the ethanol binge using flow cytometry. Compared with pair-fed mice, the percentage of Ly6G^+^CD11b^+^ neutrophils significantly increased with ethanol exposure (Figure 5C; *p* = 0.002). Thus, these data suggest that an innate immune response was triggered in the lungs with ethanol exposure.

### 3.3. Ethanol Exposure Increases Lung Immune-Cell Infiltrate and Lipocalin 2 (LCN2) Gene Expression

**Figure 6:**
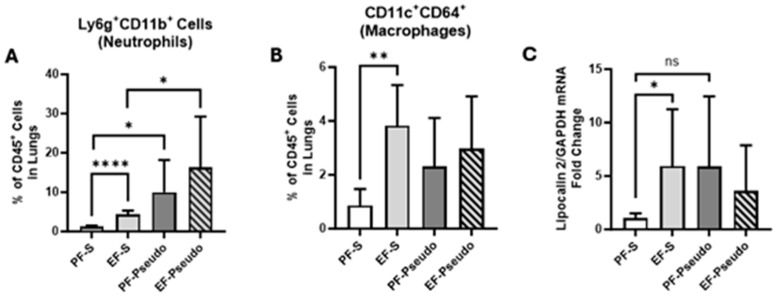
Ethanol-induced changes in lung immunity both with and without *P. aeruginosa*. (**A**) Ly6G^+^CD11b^+^ neutrophils from lung homogenate were assessed using flow cytometry. (**B**) CD11c^+^CD64^+^ macrophages from lung homogenate were assessed using flow cytometry. (**C**) LCN2 mRNA expression in lung tissue obtained via RT-PCR. There were 4–6 mice per treatment group. PF-S: control diet + intranasal saline; EF-S: ethanol diet + intranasal saline; PF-Pseudo: control diet + intranasal *P. aeruginosa*; EF-Pseudo: ethanol diet + intranasal *P. aeruginosa.* * *p* < 0.05; ** *p* < 0.01; **** *p* < 0.0001. ns: not significant.

Ethanol-induced alterations to gut microbiota have previously been shown to induce an immune response in the lungs 6 h post ethanol exposure without any infectious challenge [14]. To determine if ethanol-associated changes persisted 48 h post ethanol binge and influenced the lung’s response to an acute single *P. aeruginosa* bacterial insult, the percentages of neutrophils and macrophages in lung tissue were evaluated using flow cytometry. Both Ly6G^+^CD11b^+^ neutrophils and CD11c^+^CD64^+^ macrophages were significantly elevated in the lungs of ethanol-treated mice with nasally instilled saline vs. their pair-fed controls (Figure 6A,B; *p* < 0.0002 and *p* = 0.0017, respectively). A similar trend was also identified in ethanol-fed mice intranasally administered *P. aeruginosa*, though these findings were not statistically significant. Lipocalin 2, an iron-foraging siderophore secreted by both immune and epithelial cells in the lung, was also significantly elevated in the EF-S group vs. the PF-S mice (Figure 6C; *p* = 0.029). These data suggest that the ethanol-induced immune dysfunction in the lungs previously seen at 6 h post ethanol binge persisted up to 48 h and might influence the pulmonary immune response to an acute bacterial challenge.

## 4. Discussion

This pre-clinical study utilizing a chronic-binge ethanol-feeding mouse model that simulated the drinking patterns of people with alcohol-use disorder demonstrated that ethanol disrupted both the gut microbiome and immune responses in the gut and lung. Importantly, ethanol’s effects on pulmonary immune responses persisted up to 48 h following ethanol cessation.

Shotgun metagenomic sequencing of mouse cecal contents revealed that ethanol consumption distinctly changed bacterial populations compared with the mice not exposed to ethanol. These changes also led to a unique bacterial metabolic profile. Ethanol consumption increased the bacterial gene expression for simple-carbohydrate-generating pathways and the TCA V pathway. These changes might indicate a stress response by the gut bacteria due to ethanol-induced oxidative stress or a lack of available nutrients. The depletion of the thiamine diphosphate salvage pathway further supports this notion as thiamine is a co-factor in many metabolic processes, including the TCA and glycolysis pathways [19,34]. Interestingly, alcohol-use disorder causes a thiamine deficiency [35], and whether decreases in the bacterial salvage pathway plays a role in this deserves further study. Taken together, these changes suggest a metabolically stressed microbiome fostering a dysbiotic gut that may contribute to immune disruption in the local environment.

Our data revealed the loss of two important butyrate-producing taxa, Clostridium Cluster XIVa and *F. prausnitzii*, in the cecal microbiome during ethanol consumption. This was identified both 6 h and 48 h post ethanol exposure for Clostridium Cluster XIVa and 48 h post exposure for *F. prausnitzii*, indicating that ethanol-induced gut dysbiosis persists several days after the cessation of alcohol consumption. Microbially derived butyrate is necessary for several host-health factors, but most relevant to this work is its support of tight junctional protein complexes and the proper activation of immune responses [2,36,37,38,39]. Chronic alcohol use disrupts the intestinal barrier, inducing losses in tight junctional proteins that coincide with reductions in microbially derived butyrate [2,40,41]. Interestingly, butyrate also plays a role in lung immunity, including neutrophil recruitment and activation [42,43]. The prolonged loss of butyrate-producing bacteria identified in these studies may have played a direct role in the observed lung immune dysregulation through the consequential systemic loss of bacterially derived SCFAs. Ethanol exposure also reduced SIgA in cecal contents, reflecting a reduction in the gut luminal content. Produced by mucosal B Cells when activated by antigen-presenting dendritic cells, SIgA binds to bacteria and prevents their access to the intestinal epithelium [44,45]. A loss of SIgA and tight junctional protein integrity facilitates bacterial translocation across the intestinal mucosa.

Translocated antigens would ideally be intercepted by immune cells housed within the lamina propria of the intestine. We previously reported that ethanol exposure altered the immune-cell abundance within the lamina propria [2,14]. Cytotoxic CD8^+^ T cells, responsible for combating intracellular pathogens, were reduced within the lamina propria of the proximal colon in ethanol-exposed mice, suggesting a diminished ability to respond to a bacterial presence. Bacteria can evade the gut immune system and enter systemic circulation through several paths. Previously, we reported elevated bacteria in small intestinal Peyer’s Patches, lymphatic tissue located in the lamina propria and submucosa that collects gut antigens. Peyer’s Patches drain into mesenteric lymph nodes and the mesenteric lymph can enter the thoracic duct. If antigens are not cleared along this route, they may enter systemic circulation at the interface between the thoracic duct and subclavian vein [31,32,33]. In addition to a loss of cytotoxic T cells, we also found that CD8a^+^ dendritic cells were depleted within the mesenteric lymph nodes of ethanol-exposed mice compared with the control mice. Although systemic endotoxin was previously shown to be elevated during ethanol consumption [2,14], we also found that blood neutrophils were elevated, suggesting a systemic innate immune response, potentially from circulating antigens such as endotoxin or bacteria.

Both antigens or activated immune cells in circulation may disrupt the health and immune state of encountered organs. This premise serves as the basis for various disease conditions due to the crosstalk between the gut and various organs, such as gut–liver, gut–lung, and gut–brain axes. Although such effects can be detrimental in isolation, they could prime the organ for a “second hit”, making it vulnerable to a pathogenic challenge during subsequent exposure. In terms of the gut–lung axis, we previously reported that ethanol exposure alone induces an immune response in the lungs characterized by elevated neutrophils and markers of oxidative stress, and this response is linked to changes in the gut microbiome [13,14]. With the introduction of a pulmonary pathogen following ethanol exposure, we hypothesized that the underlying immune dysfunction in the lung initiated with ethanol exposure would compromise a proper immune response due to a bacterial challenge. Both neutrophils and macrophages were elevated in the lung tissue with ethanol exposure alone compared with the pair-fed mice. Lipocalin 2, a siderophore secreted by a variety of host cells to manage oxidative stress and deprive invading bacteria of iron, was also elevated. These findings were noted previously at 6 h and here at 48 h post ethanol binge, indicating that these changes persisted several days after the cessation of ethanol consumption with or without an intranasal inoculation of *P. aeruginosa*.

Taken together, these data support previous studies showing that ethanol-associated disruptions to the gut microbiome and immune environment are not isolated to the gut but can also impact other organ systems. Bacterial antigens taking advantage of a weakened immune response and/or weakened gut barrier can initiate a systemic immune response, demonstrated here as an elevated neutrophil presence in circulating blood and immune responsiveness in the lungs. These changes persisted despite ethanol cessation and increased the susceptibility to a pulmonary infection. Interestingly, our findings also highlighted that the gut–lung axis is not one-directional. Nasal inoculation with bacteria alone augmented gut microbial diversity compared with the pair-fed cohorts. These findings were also associated with a loss of cecal SIgA. However, it is not currently clear if the shifts in gut microbial populations were the result, or resulted in the loss, of SIgA in the cecal environment.

Although these findings offer a promising insight into how the gut–lung axis can influence host responses to infection, there were study limitations. Although an insight into a mechanistic pathway for immune dysfunction was identified within the systemic immune response, these findings were mostly associative and not causative. Future studies with models to assess for aspiration or an oral microbiome would be of interest. Although we identified alterations to the T cell presence in the lungs and intestine, other markers of T cell activation and T cell subsets would help to clarify what functional impact various T cell populations are imparting. Additionally, although a neutrophil presence in the lungs was the highest in the EF-Pseudo cohort, the data were not robust enough to show if they were significantly higher than the PF-Pseudo cohort. Future studies evaluating a more severe infectious model with a higher bacterial load or prolonged time course might allow such differences to be more readily apparent and observable. Additionally, a survival curve may be generated with a more acute infection to assess whether ethanol exposure decreases the survivability of severe pulmonary infections.

## 5. Conclusions

Ethanol consumption has a profound ability to influence health through multiple mechanisms. Recent research has highlighted that one such mechanism for these alterations is disruption to the gut microbial composition and metabolic processes that normally support intestinal health and barrier functions. The loss of such defenses allows bacterial antigens to translocate from the gut lumen into the systemic circulation, impacting the health of other organs. Here, we present evidence that this process leads to an elevated immune response, both systemically and in the lungs, and may influence the ability of the body to respond to subsequent bacterial challenges. Further follow-up investigations are warranted to fully characterize the mechanism and extent of the impact.

## Figures and Tables

**Table 1:** Primer sequences for RT-qPCR analysis of tissue mRNA.

Target	Primer	Sequence
*Lipocalin 2* NM-008491	*LCN2* F	TGG CCC TGA GTG TCA TGT G
	*LCN2* R	CTC TTG TAG CTC ATA GAT GGT GC
*GAPDH* NM_001289726	*GAPDH* F	AGG TCG GTG TGA ACG GAT TTG
	*GAPDH* R	TGT AGA CCA TGT AGT TGA GGT CA

**Table 2:** Primer sequences for RT-qPCR analysis of cecal bacteria gDNA.

Target	Primer	Sequence
Clostridium Cluster XIVa (CCXIVa) NR_104700	CCXIVa F	ACT CCT ACG GGA GGC AGC
	CCXIVa R	GCT TCT TAG TCA GGT ACC GTC AT
*Faecalibacterium prausnitzii* NR_028961	*FPrau* F	AGA TGG CCT CGC GTC CGA
	*FPrau* R	CCG AAG ACC TTC TTC CTC C
*Pseudomonas aeruginosa* NC_002516	*Pseudo* F	CCT GAC CAT CCG TCG CCA CAA C
	*Pseudo* R	CGC AGC AGG ATG CCG ACG CC
Universal Bacterial 16s	Universal F	ACT CCT ACG GGA GGC AGC AG
	Universal R	ATT ACC GCG GCT GCT GG

## Data Availability

Data supporting the reported results can be requested by writing to the corresponding author.
